# Prognostic Role of Inflammatory Indices and Real-World Outcomes in HER2-Positive Metastatic Breast Cancer Treated with Trastuzumab Emtansine

**DOI:** 10.3390/diagnostics16111746

**Published:** 2026-06-05

**Authors:** Taliha Güçlü Kantar, Tolga Doğan, Semra Taş, Bedriye Açıkgöz Yıldız, Gamze Serin Özel, Ceren Mordağ Çiçek, Ahmet Ali Kantar, Burcu Yapar Taşköylü, Atike Gökçen Demiray, Tarık Şengöz, Özgür Tanrıverdi, Arzu Yaren, Gamze Gököz Doğu

**Affiliations:** 1 Department of Medical Oncology, Denizli State Hospital, Denizli 20070, Türkiye; dr_tolgadogan94@yahoo.com; 2 Department of Medical Oncology, School of Medicine, Pamukkale University, Denizli 20070, Türkiye; semratasdr@gmail.com (S.T.); bedriyeacikgoz09@gmail.com (B.A.Y.); gamze__239@hotmail.com (G.S.Ö.); cerenmordag@gmail.com (C.M.Ç.); drburcuyapar@gmail.com (B.Y.T.); gokcenakaslan@gmail.com (A.G.D.); arzu_yaren@yahoo.com (A.Y.); ggd2882@gmail.com (G.G.D.); 3 Department of Nuclear Medicine, School of Medicine, Pamukkale University, Denizli 20070, Türkiye; drahmetalikantar@gmail.com (A.A.K.); tsengoz@pau.edu.tr (T.Ş.); 4 Department of Medical Oncology, School of Medicine, Muğla University, Mugla 48000, Türkiye; dr.ozgur.tanriverdi@gmail.com

**Keywords:** T-DM1, HER2-positive metastatic breast cancer, inflammatory indices, neutrophil-to-lymphocyte ratio, CRP/albumin ratio, real-world outcomes

## Abstract

***Background and Objectives:*** Reliable pretreatment biomarkers to guide treatment selection in HER2-positive metastatic breast cancer (mBC) remain an unmet need. Systemic inflammatory indices derived from routine blood tests have emerged as accessible prognostic markers. This study evaluated the prognostic value of inflammation-based indices in patients with HER2-positive mBC treated with trastuzumab emtansine (T-DM1). ***Materials and Methods:*** In this retrospective single-center cohort study, 50 patients with HER2-positive mBC treated with T-DM1 in the second-line setting were analyzed. Overall survival (OS) and progression-free survival (PFS) were estimated using the Kaplan–Meier method. ROC analysis assessed the prognostic performance of the CRP/albumin ratio (CAO), neutrophil-to-lymphocyte ratio (NLR), platelet-to-lymphocyte ratio (PLR), and systemic immune-inflammation index (SII). Variables associated with PFS were further evaluated using multivariable Cox regression. ***Results:*** The median follow-up was 46 months. Median OS from initial diagnosis and median PFS from T-DM1 initiation were 96 and 7 months, respectively. Metastatic pattern (*p* = 0.010), CNS involvement at T-DM1 initiation (*p* = 0.025), liver metastasis (*p* = 0.041), and best radiologic response (*p* < 0.001) were associated with PFS. ROC analysis showed modest discrimination (CAO AUC 0.694, NLR 0.658, PLR 0.646, and SII 0.653). In multivariable analysis, best radiologic response to T-DM1 was strongly associated with progression risk and appeared to reflect treatment sensitivity rather than acting as a pretreatment predictor. ***Conclusions:*** T-DM1 provided meaningful disease control in this real-world cohort. Treatment response was the main determinant of progression, while baseline inflammatory markers offered modest complementary prognostic value. These findings may aid patient selection for T-DM1, particularly in settings with limited access to trastuzumab deruxtecan.

## 1. Introduction

HER2-positive breast cancer is a biologically aggressive subtype defined by amplification or overexpression of the human epidermal growth factor receptor 2 (HER2) and, historically, an unfavorable prognosis in the metastatic setting [1]. The sequential integration of HER2-targeted agents over the past two decades has substantially transformed patient outcomes, converting metastatic HER2-positive breast cancer into a disease characterized by prolonged survival and multiple effective lines of therapy [2,3].

Trastuzumab emtansine (T-DM1) is an antibody–drug conjugate (ADC) that combines the HER2-targeting specificity of trastuzumab with the potent cytotoxic microtubule inhibitor DM1, via a stable thioether linker [4]. The landmark EMILIA phase III trial established the superiority of T-DM1 over lapatinib plus capecitabine in patients with previously treated HER2-positive advanced breast cancer, demonstrating significant improvements in both progression-free survival (PFS) and overall survival (OS) [5]. Subsequently, the TH3RESA trial confirmed the clinical activity of T-DM1 in the third-line and beyond setting [6]. Real-world series have subsequently confirmed its clinical activity across diverse patient populations, including pertuzumab-pretreated patients [7,8,9,10,11].

The landscape of HER2-directed therapy has evolved significantly with the approval of trastuzumab deruxtecan (T-DXd), a next-generation HER2-targeting ADC. The DESTINY-Breast03 phase III trial demonstrated clear superiority of T-DXd over T-DM1 as second-line therapy for HER2-positive mBC, with a 12-month PFS rate of 75.8% versus 34.1%, and T-DXd has subsequently become the preferred second-line standard of care in major international treatment guidelines [12,13]. However, the clinical relevance of T-DM1 has not been extinguished in global clinical practice. In numerous healthcare systems across Europe, Asia, the Middle East, and Latin America, reimbursement restrictions continue to limit routine access to T-DXd, making T-DM1 a highly relevant, guideline-concordant therapeutic option for many patients treated in daily practice [14,15].

Cancer-related systemic inflammation is increasingly recognized as an important determinant of disease progression, therapeutic resistance, and survival in solid tumors [16,17]. Composite hematologic indices—including the neutrophil-to-lymphocyte ratio (NLR), platelet-to-lymphocyte ratio (PLR), systemic immune-inflammation index (SII), and systemic inflammation response index (SIRI)—capture the dynamic interplay between pro-tumorigenic myeloid elements and cytotoxic lymphocyte-mediated antitumor immunity [18,19]. High NLR is associated with a protumorigenic immunosuppressive microenvironment, increased risk of metastatic spread, and impaired response to cytotoxic and targeted therapies in multiple solid tumor types [20,21]. Similarly, nutritional and acute-phase markers, such as albumin and C-reactive protein (CRP), reflect systemic inflammatory burden and metabolic reserve [22]. The CRP/albumin ratio (CAO) integrates acute-phase inflammatory response with nutritional status and has demonstrated prognostic utility in gastric, colorectal, and lung cancers [23,24]. The prognostic nutritional index (PNI), originally developed to assess perioperative nutritional risk in gastrointestinal surgery, has similarly shown prognostic relevance in various malignancies [25]. Emerging evidence suggests that inflammation-based indices may also stratify outcomes in HER2-positive metastatic breast cancer patients receiving T-DM1 [26,27,28].

Specifically, regarding T-DM1, Li et al. demonstrated that a composite score incorporating LDH and the derived NLR (dNLR) was independently predictive of OS in HER2-positive advanced breast cancer patients treated with T-DM1 [26]. Şahin et al. reported that the pan-immune-inflammation value (PIV) was significantly associated with worse outcomes in a Turkish T-DM1-treated cohort [27]. Moreover, tumor biomarker analyses from the MARIANNE trial indicated that high baseline HER2 extracellular domain levels and certain immune gene expression profiles correlated with T-DM1 efficacy [28]. Collectively, these data support the hypothesis that pretreatment inflammatory status modulates the efficacy of ADC-based therapy, potentially through effects on the tumor microenvironment, antibody-dependent cellular cytotoxicity (ADCC), and drug distribution within tumor tissue.

The novelty of the present study lies in the simultaneous evaluation of a broad panel of routinely available inflammation-based and immune-nutritional indices in patients with HER2-positive metastatic breast cancer receiving T-DM1 in a real-world setting. Previous biomarker studies in this context have generally focused on single markers or selected composite scores; therefore, assessment of CAO, NLR, PLR, SII, PNI, LMR, SIRI, and the De Ritis ratio within the same cohort may provide a more comprehensive view of host inflammatory and nutritional status. NLR and SII reflect neutrophil-dominant inflammation and relative lymphocyte suppression, PLR captures the contribution of platelet-mediated tumor progression and metastatic dissemination, and CAO integrates acute-phase inflammatory response with nutritional reserve.

Against this background, the present study aimed to evaluate the real-world efficacy of T-DM1 in a single-center cohort of patients with HER2-positive mBC, and to investigate the prognostic significance of pretreatment inflammatory and nutritional indices for survival outcomes. Given the persistent reimbursement and availability barriers affecting access to newer HER2-directed agents in many healthcare systems, this study also sought to provide clinically actionable evidence that may help refine patient selection and optimize the use of T-DM1 in routine clinical practice.

## 2. Materials and Methods

### 2.1. Study Design and Patient Population

This retrospective single-center cohort study included 50 patients with HER2-positive mBC who received T-DM1 as second-line therapy following trastuzumab–taxane treatment in the metastatic setting at Pamukkale University Medical Oncology Department. Clinical, pathological, treatment-related, and laboratory data were retrieved from electronic medical records. Baseline characteristics were reviewed in detail, including hormone receptor status, HER2 status at diagnosis and at metastatic biopsy when available, metastatic status at initial diagnosis, prior systemic therapies, metastatic distribution, number of metastatic sites, CNS involvement, liver involvement, lung involvement, and ECOG performance status. HER2 status for study eligibility was based on metastatic disease evaluation before T-DM1 initiation, including patients with acquired HER2 positivity in metastatic biopsy despite HER2-negative primary tumors. The study was approved by the Pamukkale University Non-invasive Clinical Research Ethics Committee (approval date: 11 June 2024; approval number: 2024/11). All procedures were conducted in accordance with the 1964 Declaration of Helsinki and its subsequent amendments.

#### 2.1.1. Inclusion Criteria

Histopathologically confirmed HER2-positive breast carcinoma (IHC 3+ or FISH-amplified); age ≥ 18 years; availability of complete pretreatment laboratory parameters (complete blood count, albumin, CRP, and liver function tests); radiologic and clinical evaluation performed before T-DM1 initiation and during follow-up; and complete clinical follow-up data available for survival analysis.

#### 2.1.2. Exclusion Criteria

Concomitant second primary malignancy at T-DM1 initiation; active uncontrolled infection or acute inflammatory condition at baseline blood sampling; known hematologic disorder or major autoimmune/inflammatory disease likely to confound biomarker interpretation; severe hepatic, renal, or organ dysfunction precluding reliable biomarker interpretation; and insufficient clinical, pathological, or laboratory data.

### 2.2. Definitions of Outcomes

Progression-free survival (PFS) was defined as the interval from T-DM1 initiation to the first documentation of radiologic or clinical progression, or death from any cause. Overall survival (OS) was defined as the interval from the initial breast cancer diagnosis to death from any cause or last follow-up (diagnosis-based OS). Patients without an event were censored at the last known follow-up date. Best radiologic response to T-DM1 was assessed per institutional practice using RECIST v1.1 criteria.

### 2.3. Calculation of Inflammatory and Nutritional Indices

All indices were calculated from pretreatment laboratory values obtained within 14 days before T-DM1 initiation:

*PNI* = [*serum albumin* (g/dL) × 10] + [*peripheral lymphocyte count* (/mm^3^) × 0.005]

*CAO* = *CRP* (mg/L)/*albumin* (g/dL)

*NLR* = *absolute neutrophil count*/*absolute lymphocyte count*

*PLR* = *platelet count*/*absolute lymphocyte count*

*LMR* = *absolute lymphocyte count*/*absolute monocyte count*

*SII* = *platelet count* × *neutrophil count/lymphocyte count*

*SIRI* = *neutrophil count* × *monocyte count/lymphocyte count*

*De Ritis ratio* = *AST*/*ALT*

### 2.4. Statistical Analysis

Statistical analyses were performed using IBM SPSS Statistics for Windows, version 25.0. Inflammatory biomarkers were not included in the multivariable model due to the limited sample size and risk of overfitting. (IBM Corp., Armonk, NY, USA). Categorical variables are presented as numbers and percentages; continuous variables as mean ± standard deviation (SD) or median (range), as appropriate. Optimal cut-off values for inflammatory indices were derived using ROC curve analysis with mortality as the dichotomous outcome; the Youden index was used to identify the best cut-off point. Survival distributions were estimated by the Kaplan–Meier method and compared using the log-rank test. Variables showing clinical relevance or statistical significance (*p* < 0.10) in univariable analyses were entered into a multivariable Cox proportional hazards regression model for PFS. A two-tailed *p*-value < 0.05 was considered statistically significant.

For the multivariable Cox regression model, variables were selected according to clinical relevance and/or statistical significance in univariable analyses. Because of the limited sample size and number of progression events, model complexity was intentionally restricted to reduce the risk of overfitting, and the resulting estimates should be interpreted as exploratory rather than definitive.

## 3. Results

### 3.1. Patient Characteristics

The cohort comprised 50 women with HER2-positive mBC. The median age was 49 years (range 30–74); 56.0% were aged ≤50 years and 52.0% were postmenopausal. Invasive ductal carcinoma was the predominant histologic type (94.0%), and hormone receptor co-positivity was present in 70.0% of patients. Ki-67 was ≥20% in 80.0% of cases, reflecting a predominantly high-proliferative tumor biology. Metastatic disease was present at initial diagnosis in 38.0% of patients; the remainder developed distant metastases during follow-up. The majority had combined bone and visceral metastatic involvement (66.0%), while CNS metastasis was documented at baseline in 12.0% and liver metastasis in 20.0% of patients. Complete sociodemographic and clinical characteristics are presented in Table 1.

### 3.2. Prognostic Utility of Inflammatory Indices

**Figure 1:**
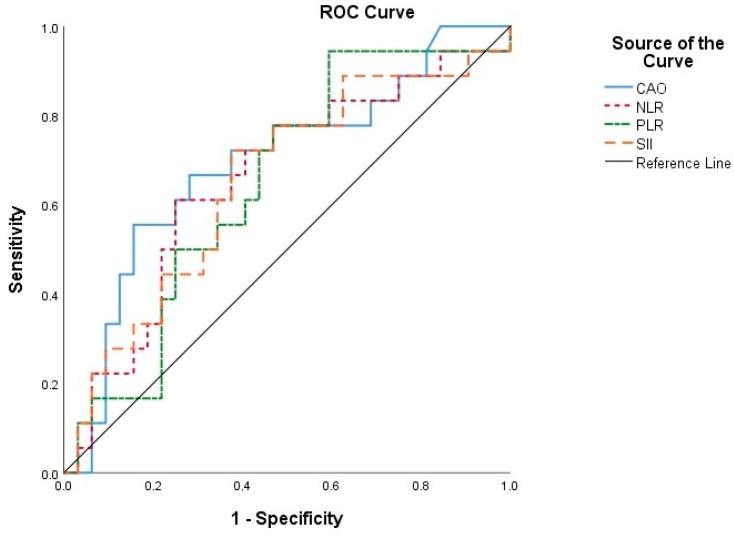
ROC Curve analysis of inflammatory indices for predicting survival outcomes.

ROC curve analysis demonstrated modest but statistically significant discriminatory performance for mortality for CAO (AUC 0.694; optimal cut-off ≥1.03; sensitivity 66.7%, specificity 65.6%; *p* = 0.024), NLR (AUC 0.658; cut-off ≥ 3.21; *p* = 0.042), PLR (AUC 0.646; cut-off ≥ 171.62; *p* = 0.045), and SII (AUC 0.653; cut-off ≥ 798,274; *p* = 0.043) (Table 2) (Figure 1). In contrast, LMR, PNI, SIRI, and the De Ritis ratio did not reach statistical significance. In Kaplan–Meier analyses, none of the inflammatory indices achieved a statistically significant difference in OS or PFS when patients were stratified by cut-off values, although numerically shorter survival was consistently observed in patients with elevated inflammatory markers.

### 3.3. Prognostic Factors for Progression-Free Survival

In log-rank analyses, patients with bone-only or predominantly visceral metastatic involvement had significantly longer PFS compared with those with combined bone and visceral disease (median 27 vs. 19 vs. 6 months, respectively; *p* = 0.010). CNS metastasis at T-DM1 initiation was associated with markedly shorter PFS (median 4 vs. 9 months; *p* = 0.025), as was liver metastasis (median 3 vs. 9 months; *p* = 0.041). In multivariable Cox regression, best radiologic response to T-DM1 was strongly associated with PFS, reflecting treatment sensitivity rather than pretreatment prognostic prediction (HR for progressive disease vs. complete response: 32.16, 95% CI: 7.05–146.67; *p* < 0.001), while metastatic pattern, CNS metastasis, and liver metastasis did not retain statistical significance after adjustment.

### 3.4. Survival Outcomes

At a median follow-up of 46 months (range 7–255 months), the median OS for the overall cohort was 96 months (95% CI: 26.3–165.7), with 2-year and 5-year OS rates of 95.8% and 71.5%, respectively. In addition, to improve comparability with pivotal T-DM1 trials and real-world series, OS from T-DM1 initiation was also calculated, yielding a median OS of 30.1 months. The median PFS after T-DM1 initiation was 7 months (95% CI: 4.1–9.9), with 2-year and 5-year PFS rates of 22.4% and 7.5%, respectively. Detailed OS and PFS comparisons stratified by clinical and biomarker variables are presented in Table 3 and Table 4.

Best radiologic response to T-DM1 had the most pronounced impact on outcome. Patients achieving complete response had a median PFS of 25 months (2-year PFS: 100%), compared with 8 months in partial responders, 5 months in those with stable disease, and only 2 months in patients with primary progression (*p* < 0.001). This association remained the strongest independent predictor of progression risk in multivariable analysis (Table 5).

## 4. Discussion

In this real-world single-center cohort of patients with HER2-positive mBC treated with T-DM1, the median PFS was 7 months. Although the observed PFS appears lower than the 9.6 months reported in the EMILIA trial, it is comparable to 6.2 months observed in the TH3RESA study [5,6] and broadly consistent with multiple real-world series that have consistently reported median PFS values in the range of 5–9 months [7,8,9,29,30]. Real-world populations invariably include greater clinical heterogeneity, higher metastatic burden, more heavily pretreated patients, and a broader range of performance statuses than those enrolled in pivotal trials—factors that collectively attenuate treatment efficacy estimates.

The relatively long OS observed in our cohort (96 months) should be interpreted with caution. In our study, OS was calculated from the time of initial breast cancer diagnosis rather than from T-DM1 initiation, incorporating the duration of prior treatment exposure, metastatic evolution, and disease-free intervals. This methodological difference limits direct comparability with registration trials and most published biomarker studies, which uniformly define OS from treatment initiation [5,6,7]. This caveat notwithstanding, the absolute OS figure reflects the meaningful disease control achievable in a contemporary HER2-positive mBC cohort receiving sequential targeted therapy, a finding consistent with the dramatically improved long-term survival reported in recent registry studies and meta-analyses of HER2-targeted treatment sequences [31,32].

Our findings reinforce that conventional disease- and treatment-related characteristics remain the principal determinants of outcome under T-DM1. The strong prognostic impact of combined metastatic distribution, liver metastasis, and CNS involvement in univariable analyses is consistent with the established adverse biology of high-burden visceral disease and intracranial dissemination in HER2-positive mBC [33,34]. CNS metastases are particularly common in HER2-positive disease, occurring in up to 30–50% of patients during the disease course, and are associated with substantially impaired outcomes due to limited CNS penetration of large-molecule HER2-targeted agents, including T-DM1 [35,36]. Most strikingly, best radiologic response to T-DM1 emerged as the dominant and independent predictor of progression risk, with hazard ratios escalating steeply across response categories—from 3.86 in partial responders, to 11.44 in patients with stable disease, and 32.16 in those with primary progression. This underscores that intrinsic treatment sensitivity and early tumor control are central determinants of durable clinical benefit from T-DM1 [37].

Resistance to T-DM1 is multifactorial and incompletely understood. Proposed mechanisms include HER2 receptor downregulation or truncation, impaired intracellular drug trafficking and lysosomal processing, upregulation of multidrug resistance transporters such as MDR1/P-glycoprotein, and alterations in microtubule dynamics conferring resistance to the DM1 payload [38]. Tumor heterogeneity and clonal evolution under HER2-targeted selection pressure may further compound acquired resistance. These biological insights underscore the finding that treatment response constitutes the strongest predictor of continued benefit and support the rationale for early treatment reassessment and switching in patients exhibiting primary resistance.

At the same time, our results provide evidence that systemic inflammatory biomarkers may offer complementary prognostic information in patients receiving T-DM1. Among the evaluated indices, CAO, NLR, PLR, and SII achieved statistically significant, albeit modest, discriminatory ability for mortality in ROC analysis, with AUC values ranging from 0.646 to 0.694. These findings are biologically plausible: elevated NLR and SII reflect neutrophil-dominated immunosuppression and a pro-inflammatory tumor microenvironment that may promote tumor progression, immunoevasion, and reduced responsiveness to ADC therapy [18,19,20]. Elevated CAO captures both the acute-phase inflammatory response and protein-calorie malnutrition, which may further compromise drug tolerance and treatment continuity [22,23,39]. The immunological basis for the prognostic value of these indices in HER2-positive disease is further supported by the known role of tumor-infiltrating lymphocytes (TILs) and immune microenvironmental composition as predictors of both prognosis and response to HER2-targeted therapy [40,41].

Importantly, LMR, PNI, SIRI, and the De Ritis ratio did not achieve significant discriminatory performance in our cohort. The failure of LMR and PNI to reach significance may relate to the overall favorable performance status of our patients—given that 74.0% had an ECOG PS of 0—which may have attenuated the range of variation in lymphocyte and albumin values necessary to discriminate outcomes. In larger and more heterogeneous populations, these markers have demonstrated prognostic utility [25,42]. The De Ritis ratio, a marker of hepatocellular injury, is perhaps more informative in the context of primary liver disease or hepatotoxic regimens, and its non-significance in our cohort is consistent with the relatively low prevalence of liver metastasis.

The present study carries particular relevance in the context of global treatment accessibility. While DESTINY-Breast03 has established T-DXd as the preferred second-line therapy for HER2-positive mBC, reimbursement restrictions significantly limit its routine use in many healthcare systems, including in Türkiye, Southern Europe, and across Asia and Latin America [12,13,14,15]. In such contexts, T-DM1 remains not merely a historical comparator, but an active and appropriate therapeutic option for the majority of eligible patients in daily practice. Our observation that a meaningful proportion of patients achieved complete or partial response to T-DM1—with complete responders demonstrating a median PFS of 25 months—underscores that substantial clinical benefit remains achievable with this agent when patients are appropriately selected. This interpretation is supported by recent real-world evidence from Italy, the United Kingdom, Türkiye, and China, as well as by multicenter analyses in pertuzumab-pretreated patients, which collectively demonstrate that T-DM1 retains meaningful activity across heterogeneous real-world populations [7,8,9,10,11,29,30].

From a practical standpoint, our findings suggest that integrating readily available baseline clinical features—particularly metastatic pattern, liver involvement, and CNS status—with simple inflammatory biomarkers may help clinicians identify patients most likely to derive benefit from T-DM1, especially when access to T-DXd is restricted. The routine availability of NLR, PLR, SII, and CRP/albumin ratio in standard pre-treatment blood work, their low cost, and the absence of any requirement for additional testing render these markers immediately implementable in routine oncology practice in low- and middle-resource settings. Future prospective studies should examine whether a composite scoring system incorporating these markers can achieve superior predictive accuracy compared with individual indices alone [43,44]. From a clinical implementation perspective, the AUC values observed for CAO, NLR, PLR, and SII (range 0.646–0.694), while modest in absolute terms, carry practical significance when interpreted in the context of routine oncology practice. These indices require no additional testing beyond standard pre-treatment blood work, incur negligible cost, and can be calculated within minutes at the point of care. In settings where T-DXd is unavailable due to reimbursement barriers, clinicians face the pragmatic challenge of identifying which patients are most likely to benefit from T-DM1 without access to expensive molecular profiling tools. In this context, a CAO cut-off of ≥1.03 or an NLR cut-off of ≥3.21 could serve as simple, objective flags to prompt more intensive early response assessment—for example, earlier restaging imaging at 6 rather than 12 weeks—or to inform shared decision-making discussions regarding the probability of meaningful disease control. Composite scoring approaches that combine two or more of these indices, potentially alongside clinical variables such as CNS status and metastatic pattern, may achieve superior discriminatory performance compared with any single marker and represent a priority for future prospective validation.

Recent biomarker studies have further emphasized the importance of integrating molecular, computational, and clinical variables for breast cancer prognostication. The study by Chakrabarty et al. highlighted the prognostic relevance of lncRNA PCAT1 in invasive breast carcinoma [45], while Li et al. proposed a fusion network-based method to identify prognostic and heterogeneous breast cancer biomarkers [46]. These studies support the broader concept that single biomarkers may be insufficient and that multidimensional risk models may better capture tumor heterogeneity.

From a mechanistic perspective, elevated inflammatory indices may represent a systemic tumor-host interaction characterized by neutrophil-mediated immunosuppression, relative depletion of lymphocyte-dependent antitumor activity, platelet-facilitated tumor cell survival in the circulation, angiogenesis, and metastatic colonization. These biological processes may contribute to tumor progression and reduced sensitivity to systemic therapy, including antibody–drug conjugates such as T-DM1.

The incremental value of inflammatory markers should be interpreted in relation to the timing of assessment. Best radiologic response is an expected post-baseline marker of treatment sensitivity, whereas inflammatory indices are available before treatment initiation. Therefore, baseline inflammatory markers may provide supportive pretreatment risk information and may help identify patients who require closer early monitoring, although they cannot replace radiologic response assessment.

The modest AUC values observed in this study indicate limited discrimination and should not be overinterpreted. In clinical practice, biomarkers with AUC values in this range are unlikely to be sufficient for independent treatment selection, but they may still have practical value when integrated into composite models that include metastatic burden, CNS involvement, liver metastasis, performance status, and treatment history.

The present study has several limitations that require acknowledgment. First, the retrospective, single-center design limits external validity and introduces the possibility of selection bias. Second, the sample size of 50 patients is relatively modest, potentially limiting statistical power for biomarker analyses. Third, inflammatory indices were assessed only at baseline; longitudinal changes during treatment that might provide additional prognostic information were not captured. Fourth, as noted above, the definition of OS from initial diagnosis rather than T-DM1 initiation limits cross-study comparability. Finally, residual confounding related to prior treatment sequences, tumor biology, and institutional practice cannot be excluded.

Despite these limitations, this study provides real-world evidence regarding T-DM1 outcomes and demonstrates the potential supplementary prognostic value of simple, inexpensive, and universally available inflammatory biomarkers. Larger, prospective, multicenter studies are warranted to validate the optimal cut-offs identified in our ROC analyses, to evaluate the dynamic prognostic contribution of inflammatory markers during T-DM1 treatment, and to define composite prognostic models that may guide individualized treatment sequencing in HER2-positive mBC.

## 5. Conclusions

T-DM1 provided clinically meaningful disease control in this real-world cohort of patients with HER2-positive mBC. Best radiologic response was the dominant and independent determinant of progression risk, with hazard ratios escalating markedly across response categories. Metastatic pattern, liver metastasis, and CNS involvement exerted significant prognostic influence in univariable analyses, though their independent effects were attenuated in multivariable modeling. Pretreatment inflammatory biomarkers—particularly CAO, NLR, PLR, and SII—demonstrated modest but statistically significant discriminatory performance for mortality, providing complementary prognostic information beyond conventional clinical factors.

In many countries, trastuzumab deruxtecan, while now the preferred second-line option in HER2-positive mBC, is not consistently accessible due to reimbursement restrictions. In such settings, T-DM1 continues to represent an important and guideline-concordant therapeutic option. Our results suggest that incorporating baseline clinical characteristics together with simple, routinely available inflammatory biomarkers may support more informed treatment decision-making and patient selection for T-DM1, particularly where access to newer agents is constrained. Multicenter studies with larger sample sizes are needed to validate these findings and to develop robust prognostic tools for T-DM1 therapy in contemporary clinical practice.

## Figures and Tables

**Table 1:** Sociodemographic and clinical characteristics of the study cohort (*n* = 50).

Variables	*n*	%
*Age*		
Mean ± SD	48.52 ± 10.41	
Median (range)	49 (30–74)	
≤50 years	28	56.0
>50 years	22	44.0
*Comorbidity*		
None	37	74.0
Present	13	26.0
*Menopausal Status*		
Premenopausal	24	48.0
Postmenopausal	26	52.0
*Histologic Type*		
Invasive ductal carcinoma	47	94.0
Other	3	6.0
*Hormone Receptor Status*		
Positive	35	70.0
Negative	15	30.0
*HER2 Status at Diagnosis*		
Positive	29	58.0
Negative (acquired HER2-positivity)	21	42.0
*Ki-67 Proliferation Index*		
<20%	10	20.0
≥20%	40	80.0
*Metastatic at Diagnosis*		
No	31	62.0
Yes	19	38.0
*Metastatic Pattern*		
Bone-only	1	2.0
Visceral-only	16	32.0
Combined (bone + visceral)	33	66.0
*Number of Metastatic Sites*		
1	9	18.0
2	25	50.0
3	13	26.0
≥4	3	6.0
*CNS Metastasis at T-DM1 Initiation*		
No	38	76.0
Yes	12	24.0
*Liver Metastasis*		
No	40	80.0
Yes	10	20.0
*Lung Metastasis*		
No	29	58.0
Yes	21	42.0
*ECOG Performance Status Before T-DM1*		
0	37	74.0
1	12	24.0
2	1	2.0
*Best Radiologic Response to T-DM1*		
Complete response	14	28.0
Partial response	22	44.0
Stable disease	4	8.0
Progressive disease	10	20.0
*CRP*		
<5 mg/L	29	58.0
>5 mg/L	21	42.0
*Radiologic Progression on T-DM1*		
No	15	30.0
Yes	35	70.0
*Mortality*		
Alive	32	64.0
Deceased	18	36.0
*PFS on T-DM1 (months)*		
Mean ± SD	10.72 ± 14.00	
Median	7	
*OS from T-DM1 initiation (months)*		
Mean ± SD	16.60 ± 16.41	
Median	30.1	
*Follow-up Duration (months)*		
Mean ± SD	64.62 ± 56.29	
Median (range)	46 (7–255)	

**Table 2:** ROC analysis: discriminatory performance of inflammatory indices for mortality.

Index	AUC	95% CI	Cut-Off	Sensitivity (%)	Specificity (%)	*p*
CAO	0.694	0.537–0.850	≥1.03	66.7	65.6	0.024 *
NLR	0.658	0.496–0.819	≥3.21	61.1	62.5	0.042 *
PLR	0.646	0.489–0.803	≥171.62	61.1	59.4	0.045 *
SII	0.653	0.491–0.814	≥798,274	61.1	62.5	0.043 *
LMR	0.581	0.406–0.755	≤3.39	66.7	65.6	0.347
PNI	0.592	0.424–0.760	≤48.62	55.6	56.3	0.284
SIRI	0.564	0.394–0.735	≥1358.77	61.1	62.5	0.455
De Ritis ratio	0.507	0.331–0.683	≥1.14	55.6	56.3	0.936

AUC, area under the curve; CI, confidence interval. * *p* < 0.05 statistically significant.

**Table 3:** Kaplan–Meier overall survival comparisons by clinical and biomarker variables.

Variable	2-Year OS (%)	5-Year OS (%)	Median OS (95% CI), Months	*p*
*Overall*	95.8	71.5	96.0 (26.3–165.7)	
*Age*				
≤50 years	96.4	70.7	96.0 (13.2–178.8)	0.569
>50 years	94.7	73.7	133.0 (57.5–208.5)	
*Metastatic Pattern*				
Bone-only	—	—	181.0 (—)	0.962
Visceral-only	100.0	48.2	52.0 (—)	
Combined	93.8	77.5	96.0 (33.8–158.2)	
*Liver Metastasis*				
No	97.1	78.0	133.0 (62.7–203.3)	0.205
Yes	90.0	45.0	52.0 (11.6–92.4)	
*CNS Metastasis at T-DM1 Initiation*				
No	97.0	74.9	133.0 (37.6–228.4)	0.744
Yes	91.7	62.5	96.0 (6.7–185.3)	
*Best Response to T-DM1*				
Complete response	100.0	83.3	— (—)	0.518
Partial response	90.9	71.4	96.0 (47.5–144.5)	
Stable disease	—	75.0	— (—)	
Progressive disease	100.0	60.0	86.0 (10.3–161.7)	
*CAO*				
<1.03	95.7	75.3	— (—)	0.193
≥1.03	95.7	68.0	86.0 (0.0–173.2)	
*NLR*				
<3.21	95.5	72.3	181.0 (43.3–318.7)	0.451
≥3.21	95.7	71.7	96.0 (43.3–148.7)	
*SII*				
<798,274	100.0	76.6	181.0 (42.4–319.6)	0.342
≥798,274	91.3	67.0	96.0 (40.6–151.4)	

Log-rank test; *p* < 0.05 statistically significant. CI, confidence interval.

**Table 4:** Kaplan–Meier progression-free survival comparisons by clinical and biomarker variables.

Variable	2-Year PFS (%)	5-Year PFS (%)	Median PFS (95% CI), Months	*p*
*Overall*	22.4	7.5	7.0 (4.1–9.9)	
*Age*				
≤50 years	22.7	—	7.0 (2.8–11.2)	0.622
>50 years	20.4	10.2	9.0 (3.0–15.0)	
*Metastatic Pattern*				
Bone-only	100.0	—	27.0 (—)	0.010
Visceral-only	29.6	29.6	19.0 (4.7–33.3)	
Combined	14.8	—	6.0 (4.5–7.5)	
*CNS Metastasis at T-DM1 Initiation*				
No	25.3	8.4	9.0 (5.7–12.3)	0.025
Yes	—	—	4.0 (0.1–7.9)	
*Liver Metastasis*				
No	21.6	8.7	9.0 (3.9–14.1)	0.041
Yes	25.0	—	3.0 (1.6–4.4)	
*Lung Metastasis*				
No	28.2	14.1	11.0 (4.0–18.0)	0.263
Yes	16.0	—	6.0 (4.6–7.4)	
*Best Response to T-DM1*				
Complete response	100.0	83.3	25.0 (—)	<0.001
Partial response	90.9	71.4	8.0 (3.8–12.2)	
Stable disease	—	75.0	5.0 (2.1–7.9)	
Progressive disease	0.0	0.0	2.0 (—)	
*CAO*				
<1.03	9.6	9.6	9.0 (6.2–11.8)	0.361
≥1.03	21.2	—	5.0 (3.0–7.0)	
*NLR*				
<3.21	23.8	17.8	12.0 (6.9–17.1)	0.096
≥3.21	16.7	—	6.0 (3.8–8.2)	
*SII*				
<798,274	25.4	12.7	9.0 (6.4–11.6)	0.689
≥798,274	21.1	—	6.0 (3.7–8.3)	

Log-rank test; *p* < 0.05 statistically significant. CI, confidence interval.

**Table 5:** Multivariable Cox regression analysis for progression-free survival.

PFS (Months)	HR (95% CI)	*p*
*Metastatic Pattern*		0.255
Bone-only	Reference	
Visceral-only	1.60 (0.15–16.31)	0.688
Combined	3.14 (0.38–25.86)	0.286
*CNS Metastasis at T-DM1 Initiation*		
No	Reference	
Yes	1.60 (0.55–4.63)	0.386
*Liver Metastasis*		
No	Reference	
Yes	1.73 (0.68–4.38)	0.242
*Best Response to T-DM1*		<0.001
Complete response	Reference	
Partial response	3.86 (1.10–13.59)	0.035
Stable disease	11.44 (2.38–54.88)	0.002
Progressive disease	32.16 (7.05–146.67)	<0.001

HR, hazard ratio; CI, confidence interval. −2 log likelihood = 180.51; *p* < 0.001.

## Data Availability

The data presented in this study are available from the corresponding author upon reasonable request.
